# Immunomodulatory Effects of Exercise in Experimental Multiple Sclerosis

**DOI:** 10.3389/fimmu.2019.02197

**Published:** 2019-09-13

**Authors:** Antonietta Gentile, Alessandra Musella, Francesca De Vito, Francesca Romana Rizzo, Diego Fresegna, Silvia Bullitta, Valentina Vanni, Livia Guadalupi, Mario Stampanoni Bassi, Fabio Buttari, Diego Centonze, Georgia Mandolesi

**Affiliations:** ^1^Synaptic Immunopathology Lab, Department of Systems Medicine, Tor Vergata University, Rome, Italy; ^2^Synaptic Immunopathology Lab, IRCCS San Raffaele Pisana, Rome, Italy; ^3^San Raffaele University, Rome, Italy; ^4^Unit of Neurology, IRCCS Neuromed, Pozzilli, Italy

**Keywords:** rehabilitation, experimental autoimmune encephalomyelitis (EAE), cuprizone (CPZ), lysolecithin (LPC), Theiler's virus-induced demyelinating disease (TMEV-IDD), microglia, T cell, astroglia

## Abstract

Multiple Sclerosis (MS) is a demyelinating and neurodegenerative disease. Though a specific antigen has not been identified, it is widely accepted that MS is an autoimmune disorder characterized by myelin-directed immune attack. Pharmacological treatments for MS are based on immunomodulatory or immunosuppressant drugs, designed to attenuate or dampen the immune reaction, to improve neurological functions. Recently, rehabilitation has gained increasing attention in the scientific community dealing with MS. Engagement of people with MS in exercise programs has been associated with a number of functional improvements in mobility, balance, and motor coordination. Moreover, several studies indicate the effectiveness of exercise against fatigue and mood disorders that are frequently associated with the disease. However, whether exercise acts like an immunomodulatory therapy is still an unresolved question. A good tool to address this issue is provided by the study of the immunomodulatory effects of exercise in an animal model of MS, including the experimental autoimmune encephalomyelitis (EAE), the Theiler's virus induced-demyelinating disease (TMEV-IDD) and toxic-demyelinating models, cuprizone (CPZ), and lysolecithin (LPC). So far, despite the availability of different animal models, most of the pre-clinical data have been gained in EAE and to a lesser extent in CPZ and LPC. These studies have highlighted beneficial effects of exercise, suggesting the modulation of both the innate and the adaptive immune response in the peripheral blood as well as in the brain. In the present paper, starting from the biological differences among MS animal models in terms of immune system involvement, we revise the literature regarding the effects of exercise in EAE, CPZ, and LPC, and critically highlight the advantages of either model, including the so-far unexplored TMEV-IDD, to address the immune effects of exercise in MS.

## Introduction

Rehabilitation is a supportive therapy increasingly required to manage symptoms and improve quality of life (QoL) in many neurological disorders, including Multiple Sclerosis (MS), the most common neurodegenerative disease affecting young adults. MS patients encounter a number of symptoms, from sensory-motor impairments to psychiatric comorbidities, which heavily impair daily life activities and make compulsory the search for effective therapies (1). Disease modifying therapies (DMTs) for MS are immunomodulatory or immunosuppressive agents designed to dampen the immune reaction that is the main trigger of the disease. Indeed, MS is likely caused by the loss of immune tolerance against myelin epitopes, leading to autoimmune attack against myelin with the consequence of demyelination, axonal damage, and neuronal loss in several brain areas through intermingled and parallel processes (2, 3). This, in turn, explains the heterogeneity of symptoms and clinical phenotypes of MS, which include two main subgroups, Relapsing Remitting (RRMS) and Progressive MS (PMS). DMTs can control disease progression, increasing life expectations, but they are not devoid of side effects (4) and, with some exceptions, their applications are limited to RRMS.

Exercise has been convincingly associated with functional recovery of several disease outcomes, such as walking (5), balance (6), and fatigue (7). Likewise, other symptoms, like depression and cognition, can be improved by exercise (7, 8). Despite increasing acknowledgment of the beneficial effects of exercise in MS, engagement of patients in rehabilitation programs is still low (9). A reason for such discrepancy stems from incomplete comprehension of exercise as a DMT. While DMTs have a specific target in MS, namely the immune system, the substrate of action of exercise is still poorly understood. In this regard, only few clinical studies have addressed exercise effects on the dysregulated immune response in MS (9). A possibility to explore this issue is to test exercise in pre-clinical models of MS, which, though with some limitations thereafter addressed, allow for deep analysis of the impact of treatments, both pharmacological and non-pharmacological, like exercise, on single pathological processes.

In this mini-review, we will briefly summarize the key elements involved in immune response in human and experimental MS to provide a general overview of the immune component involved and possibly targeted by experimental paradigms of exercise in rodents. Next, we will revise animal studies exploring the immunomodulatory action of exercise, critically highlighting possible future directions.

## Immune Players in MS Pathogenesis

Cells of both the adaptive and the innate immune response have been claimed to play a crucial role in the initiation and the progression of the disease [for an exhaustive description, we refer the reader to (10)]. Although the reasons for the expansion of autoreactive lymphocytes in lymphoid tissues in MS is still a matter of investigation and debate (2), the disease is likely triggered by the infiltration of helper CD4+, cytotoxic CD8+ T cells, and B lymphocytes, members of the adaptive immune system, into the CNS. This step is followed by the involvement of cells of the innate immune system: the activation of resident microglia and the recruitment of monocyte-derived macrophages, together with reactive astrocytes, contribute to amplify the inflammatory response, for example by releasing pro-inflammatory cytokines. Indeed, clusters of lymphocytes have been shown in both gray matter and demyelinating plaques of MS brains in the presence of activated microglia and astrocytes. Importantly, during the phases of inactivity of the disease, i.e., without reactivation of inflammatory foci, B cells, which accumulate in meningeal B-cell follicles (11, 12), are supposed to play a vital role in making the brain inflammatory reaction chronic and to support neurodegeneration in PMS.

### Animal Models of MS

An animal model is predicted to recapitulate the main pathological and clinical features of a given human disease. Different animal models of MS have been developed to account for the difficulty to reproduce the whole spectrum of pathological events occurring in MS and the different phenotypes in a unique experimental system (13).

The experimental autoimmune encephalomyelitis (EAE) is the most used to investigate immunological events in the brain (14) and consists in the administration of myelin antigens to elicit a T cell-mediated immune response in the receiving animal. Depending on species (rats or mice), strain (C57BL6 or SJL mice), type of immunization (passive or active), and immunogen (recombinant peptide or spinal cord homogenate), EAE can evolve as a relapsing-remitting (RR-EAE) or a chronic disease (CR-EAE), meaning they have different immunological patterns. The main criticism to EAE is that it reflects an immune response merely guided by CD4+ T cells (13). Other inflammatory MS pathological hallmarks, like microgliosis and astrogliosis, are well-represented.

Several viral models have been developed to account for the environmental susceptibility factors linked to MS etiopathogenesis (15). The most used is the Theiler's virus-induced demyelinating disease (TMEV-IDD) that is a model of chronic progressive MS. The Theiler's virus infects microglia/macrophages (16) as well as T cells (17), producing a kind of “natural” activation of the whole immune system and induces a chronic disease exclusively in mice. Compared to EAE, it is supposed to better reproduce the immune activation and pathological responses of human MS, even if persistent viral infection in the MS brain has not been demonstrated so far. Moreover, it is useful to study axonal damage, that in this model precedes demyelination and involves T cell recruitment (18).

The toxic-demyelinating models, namely lysolecithin (LPC) and cuprizone (CPZ), are used to study demyelination and remyelination processes and reproduce autoimmune-independent myelin damage. Lysolecithin (LPC) is a detergent supposed to be toxic for myelin. Injected directly into a specific brain area, LPC induces a focal demyelination, which triggers a fast recruitment of macrophages and microglia followed by T and B cells to repair myelin damage (19). CPZ is a copper chelator predicted to be toxic for mature oligodendrocytes (OLs) (20). Given orally, it induces a highly reproducible demyelination in several brain areas, especially the corpus callosum (CC), and a consistent activation of both microglia and astroglia (21). Histopathological damage caused by CPZ mirrors a pure primary oligodendropathy, which is found in MS lesions type-III and IV (22).

### Exercise Paradigms in Rodents

Different paradigms of behavioral interventions can be applied to rodents. Environmental enrichment (EE), consisting in rearing mice or rats in extra-size cages containing toys, running wheels, tunnels, and other devices, is considered a positive modulator of both motor and sensory systems known to modulate both synaptic activity and immunity (23, 24). In terms of translational relevance, EE refers to life-style interventions aimed to improve well-being.

Other approaches are intended to simulate the effects of physical training in humans and fall into two categories, forced (FE) and voluntary (VE) exercise (25). In FE, animals are subjected to daily repeated exercises provided by means of dedicated apparatuses, like a motorized treadmill, a swimming pool (both for endurance training) (26), or a vertical ladder (strength training) (27). VE consists in housing animals in cages endowed with a wheel to which they have free access, and is generally referred to as voluntary running-wheel (28).

The main differences between FE and VE rely on the amount of stress that FE can induce to animals (29) and the variability of VE extent, since in this case the activity is based on the animal's willingness to run. Moreover, daily and total duration of exercise as well as regimen, whether preventive or therapeutic, can affect results and must be considered when comparing different protocols.

### FE in MS Animal Models

Most of the studies addressing the effects of exercise in experimental MS, have been conducted in EAE and to a lesser extent in CPZ and LPC, while none has been performed in TMEV-IDD. Such studies have focused on immunomodulation and/or regulation of neurotrophic factors in the brain as underlying putative mechanisms (30) (Table 1). Other studies, here not revised, have focused on muscular or neuroendocrine effects of exercise (31–33).

**Table 1:** Summary of referenced studies addressing immunomodulatory and neuroprotective effects of exercise in MS animal models.

		**MS animal model**	**Effects of exercise on adaptive immune system**	**Effects of exercise on innate immune system**	**Effects of exercise on brain inflammation**	**Effects of exercise on brain neurotrophins**	**Other effects of exercise or only effects on clinical score**
Forced exercise	Preventive	EAE	37; 38; 39; 42; 43;44	37; 38; 39; 47	36; 37; 42; 43; 44	36; 37; 42; 43; 44	34; 35
		CPZ		40		40	41
	Therapeutic	EAE				45; 46	
Environmental enrichment	Preventive	EAE	50; 51		49		48; 50
		LPC					48
Voluntary exercise	Preventive	EAE	51; 52; 53; 54	53; 54			
		CPZ		56			
		LPC		55			
	Therapeutic	EAE				46	

The first reports showed that 10-day treadmill training initiated soon after EAE induction was effective in reducing clinical disability in RR-EAE rats (34, 35). This result was not replicated by another group, who failed to detect significant changes of TNF concentrations but found a significant increase of NGF in exercise-EAE brains (36). Later on, immune effects of running treadmill (endurance training) were studied in comparison to climbing ladder (strength training) (37). Both protocols, initiated 2 weeks before EAE induction and for a total of 4 weeks, attenuated disease course and reduced brain levels of pro-inflammatory cytokines, IFN-γ, IL1-β, and IL-17. During acute and chronic phases, strength training, and endurance training attenuated the innate immune response of dendritic cells in spleen and in secondary lymphoid organs, respectively. Interestingly, strength training induced upregulation of markers of regulatory T cell (Tregs) in splenocyte population, suggesting that this T cell subpopulation could be involved in the beneficial effects of exercise on EAE pathology and that different types of exercise could induce distinctive immunomodulatory activities (37).

More recently, two elegant studies have corroborated the idea that exercise-mediated beneficial effects on EAE course occur through a peripheral immunomodulation (38, 39). Adoptive transfer of T cells from donor EAE mice undergoing a preventive treadmill training into sedentary recipient mice induced a less severe EAE disease course in sedentary recipient mice, compared to mice receiving lymphocytes taken from sedentary donor EAE mice (38) or undergoing a less intensive training (39). The induction of EAE by transferring T cells from donor sedentary-mice into trained mice did not ameliorate clinical and pathological aspects of the disease, suggesting the lack of direct neuroprotective effect of exercise (38). Moreover, response to acute bacterial infection was not affected by exercise, indicating that training did not influence the innate immune response (39).

FE has been tested in CPZ model (40, 41). Two treadmill-based protocols, the high-intensity interval training (HIIT) and the low-intensity continuous training (LICT), started 4 weeks before CPZ feeding, reduced motor deficits of CPZ-injured animals and increased the mRNA of neurotrophic factors in the hippocampus. Moreover, HIIT significantly increased hippocampal microglia density, improving neuroinflammation, likely as a consequence of amount of the stress induced by the FE. However, the technical detection of microglia is questionable and weakened the result (40). Moreover, treadmill and swimming FE given during and after a 12 week-CPZ treatment significantly improved locomotor activity (41).

More consistent data have been obtained with swimming. A preventive swimming training started 4 weeks before EAE induction and maintained until 10 days post-immunization (dpi) attenuated EAE severity, without changing the number of infiltrating mononuclear cells and promoting a reduced and an increased pro-inflammatory environment in brain and in spinal cord of exercising mice, respectively (42). Swimming significantly increased BDNF levels in spinal cord and brain homogenates of EAE mice. These effects were observed at 14 dpi, soon after the disease onset. Thereafter, the same group analyzed long-term effects of such preventive swimming protocol, showing that it significantly reduced the number of spinal cord infiltrating CD4+ and CD8+ T cells and B cells at 42 dpi (43).

More recently, preventive high-intensity swimming has been shown to reduce EAE disability and the number of spinal cord infiltrating cells (44). Exercise upregulated Tregs in both spinal cord and lymph nodes, reduced IFN-γ and IL-17 levels and increased the amount of TGF-β and IL-10 in spinal cord, confirming that exercise bears neuroprotection through immunomodulatory activity. Interestingly, exercise increased BDNF levels exclusively in the brain and not in the blood, suggesting that the rise of neurotrophin levels was independent of immunomodulation.

Therapeutic treadmill exercise has also been tested. In CR-EAE, exercise which started at 20 dpi for 4 weeks improved hippocampal demyelination and neurogenesis, memory function, and BDNF levels (45). In rat RR-EAE, both VE and treadmill exercise delivered during the remission period after initial disease onset, did not induce significant effects on disease course and levels of hippocampal BDNF (46). However, the immune response was not assessed in any of these studies.

Finally, a recent study deserves attention. Bernardes et al. (47) faced, for the first time, the “preconditioning” role of exercise on the beneficial effects of two DMTs, glatiramer acetate (GA) and dimethyl fumarate (DMF). Preventive treadmill training did not ameliorate both the severity and the histopathological hallmarks of the disease compared to sedentary mice, confirming that such kind of exercise can induce variable responses in EAE. However, exercise associated to drugs administered therapeutically during the acute phase of the disease reinforced the beneficial effects of DMTs, providing further attenuation of spinal cord astrogliosis in combination with GA and increasing microgliosis and synaptic inputs at motoneurons in spinal cord of DMF-treated mice. A slightly significant amelioration of clinical score was observed in DMF/exercise EAE mice.

### EE and VE in MS Animal Models

Few studies have tested the effects of EE in MS models. The first paper showed that EE initiated the day of EAE induction improved motor dysfunction and induced mobilization of precursor cells in demyelinating areas. The latter effect was also confirmed in LPC injured-mice. However, the anti-inflammatory action of EE was not assessed (48). The beneficial effects of EE on EAE clinical course were later confirmed by studies assessing the pre-conditioning role of EE (49, 50). EAE mice reared in EE condition before immunization showed a slight reduction of inflammatory infiltrates in cortex, but not in spinal cord, without changes of IL1-β levels in spinal cord (49). Recently, Xiao et al. (50) strikingly demonstrated the immunomodulatory action of preventive EE. EE significantly attenuated EAE-induced thymic retraction, reduced the fraction of T helper type-1 (Th1) cells in the thymus and the spleen in favor of Tregs and the expression of the early activation marker CD69 on CD4+ cells. Adoptive transfer of thymocytes from EE-mice into donor sedentary mice induced an attenuated disease, highlighting the role of thymic population in EE-mediated immunomodulation. Finally, EAE induction in mice lacking the corticosteroid receptor in thymocytes, prevented EE-beneficial effect, suggesting the involvement of the pituitary-adrenal axis.

VE has been invariably associated with recovery of motor function in EAE mice, but its immunomodulatory action has been faintly investigated, thus providing contrasting results. Indeed, VE started the day of EAE induction (51) did not alter both infiltrating cells, detected by histological techniques, and demyelination extent in the striatum and the spinal cord of EAE-exercise mice, despite the improved disease course. Based on the finding that exercise recovered synaptic abnormalities in these mice, the authors argued that VE exerted a direct neuroprotective effect, although not assessing the levels of neurotrophic factors. Conversely, through a different histological approach, another group found significant reduction of spinal cord cellular infiltration in EAE mice undergoing VE (52). In these studies, animals were reared in running wheel cages, allowing *ad libitum* free access to wheel. Notably, 1 h per day of free access to running wheel was found to delay disease onset, without affecting disease severity, and to reduce microgliosis and T cell infiltration in spinal cord (53, 54).

VE has been tested in demyelinating toxic models. VE initiated soon after LPC injection in spinal cord induced an early shift from a pro-inflammatory to an anti-inflammatory phenotype of microglia/macrophages, which, by removing myelin debris, allowed the proliferation of OPCs (55). The combination of exercise and clemastine, an anti-muscarinic drug with OPC pro-differentiating properties, resulted in an additive increase of new OLs, corroborating the hypothesis that exercise created a permissive environment for remyelination.

Recently, we have investigated the effects of VE in CPZ (56). Exercise started together with CPZ feeding improved motor dysfunction associated with the toxicant treatment. After 3 weeks, VE induced an early protection against myelin loss together with lessened microglia activation in CC, an area highly sensitive to CPZ. At later stages, astrogliosis was significantly attenuated as well. We proposed that exercise could exert a direct protection on myelin that, in turn, might limit microglia proliferation and activation in the damaged white matter area, with the consequence of a reduced recruitment of new OLs during the late phase of CPZ feeding and increased myelin content.

## Conclusions and Perspectives

Understanding the DMT potential of exercise in human and experimental MS is a challenging topic, which is gaining increasing interest. Nevertheless, the lack of standardization of both exercise protocols and evaluation scales in human studies does not allow a comprehensive analysis of the effects of rehabilitation in MS subjects (9). Rehabilitative research applied to animal models indicates that the effects of exercise may depend on several factors, including the length, the type and the regimen of exercise as well as the amount of stress induced. These data, translated to clinical practice, further support the idea of a personalized exercise therapy for MS patients to better foster functional recovery (57).

**Figure 1:**
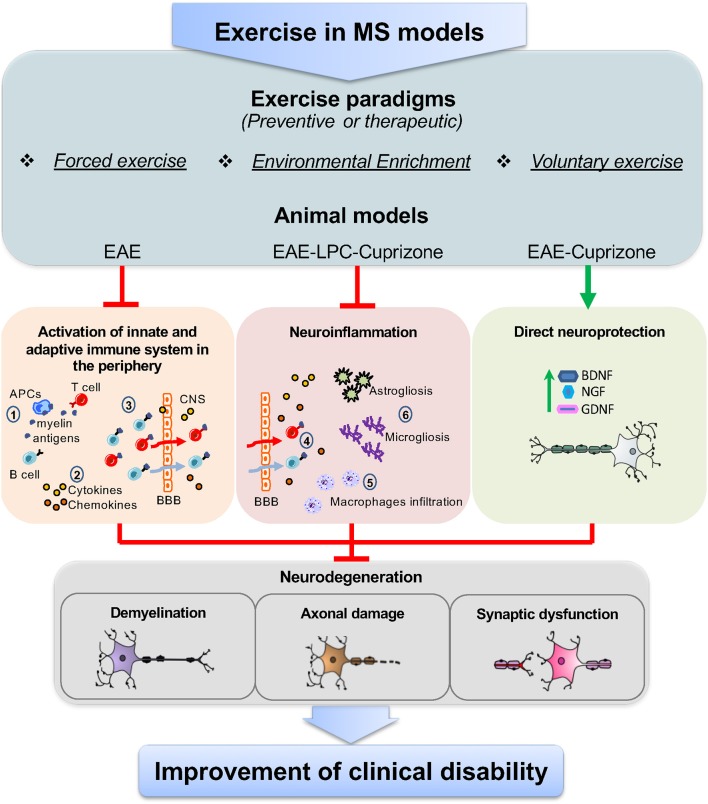
Targets of exercise in MS animal models. The use of different exercise paradigms, including forced exercise, environmental enrichment, and voluntary exercise applied to animal models of MS, such as EAE, and the toxic-demyelinating models LPC and CPZ, has highlighted that the immunomodulatory effects of exercise can occur through parallel mechanisms together with a direct neuroprotective action. Studies from EAE mice have shown that, in the periphery, exercise reduces the immune-specific response of antigen presenting cells (APCs) against myelin epitopes, thereby limiting T and B cells activation (1). In addition to this, exercise lowers the levels of soluble inflammatory markers, namely cytokines and chemokines, as the consequence of the reduced activation of T and B cells and/or the modulation of lymphocyte subpopulations (2). Moreover, exercise has been proven to restrain the number of T and B cells that cross the blood brain barrier (BBB) (3). This, in turn, results in the reduced number of infiltrating T and B cells in the brain (4) and the attenuation of typical neuroinflammatory hallmarks, like macrophage infiltration (5), astrogliosis and microgliosis (6). However, data from LPC and CPZ models show that exercise can directly modulate brain resident immune cells (astrocyte, microglia, and macrophages). Concurrently, as highlighted in both EAE and CPZ, exercise activates neuroprotective pathways, through the increase of neurotrophins, like BDNF, NGF, and GDNF, which can restore neuronal functioning. As a result of the above mechanisms, exercise attenuates hallmarks of neurodegeneration in the brain of MS animal models, like demyelination, axonal damage, and synaptic dysfunction, leading to the improvement of clinical disability.

Overall, pre-clinical studies performed in different MS models suggest that exercise may involve multiple, parallel, and non-exclusive immunoregulatory mechanisms (Figure 1). On one hand, data from EAE show that preventive FE has a DMT potential, likely due to attenuation of T cell response and brain infiltration, though direct neuroprotective mechanisms mediated by neurotrophins are also likely involved. However, since exercise-mediated immune effects have been characterized mostly in CR-EAE, it cannot be excluded the possibility that exercise activates different immune mechanisms in RR-EAE. On the other hand, studies in LPC and CPZ suggest that exercise can have microglia-targeted effects independent of peripheral immunomodulation (55, 56). Yet, the importance of demyelinating models and the so far unexplored TMEV-IDD for the study of exercise-DMT potential is still underestimated.

Exercise appears to exert a pre-conditioning effect to the development and/or progression of the disease, raising two clinically relevant and interconnected questions. The first refers to the immunomodulating role of a therapeutic regimen of exercise and the possibility that exercise activates different mechanisms respect to the preventive regimen. The second regards the association of exercise to currently approved DMTs and the possible synergistic effects on pathological hallmarks of the disease. Both issues are barely addressed.

Finally, we envisage the importance of understanding in depth the immunomodulatory action of EE and VE, since they are reported to exert a control on immune and nervous systems in healthy condition (23, 24, 50) and are devoid of stress effects that can be induced in animals during FE. Moreover, VE is considered a rewarding activity (58), which, on a clinical level, would contribute to increase QoL in MS patients.

## Author Contributions

AG, DC, and GM conceived and designed the manuscript. All the other authors made significant contributions, reviewed, and approved the manuscript.

### Conflict of Interest Statement

The authors declare that the research was conducted in the absence of any commercial or financial relationships that could be construed as a potential conflict of interest.
